# Housing-related challenges during COVID-19 pandemic among urban poor in low- and middle-income countries: A systematic review and gap analysis

**DOI:** 10.3389/fpubh.2022.1029394

**Published:** 2022-09-23

**Authors:** Shubhankar Dubey, Krushna Chandra Sahoo, Girish Chandra Dash, Mili Roopchand Sahay, Pranab Mahapatra, Debdutta Bhattacharya, Mariam Otmani del Barrio, Sanghamitra Pati

**Affiliations:** ^1^Health Technology Assessment in India, ICMR-Regional Medical Research Centre, Bhubaneswar, India; ^2^Department of Psychiatry, Kalinga Institute of Medical Sciences, Bhubaneswar, India; ^3^UNICEF/UNDP/World Bank/WHO Special Programme for Research and Training in Tropical Diseases (TDR), World Health Organization, Geneva, Switzerland

**Keywords:** housing, urban poor, opportunities, challenges, COVID-19, LMIC

## Abstract

The abysmal health of the urban poor or slum dwellers was attributed to structural inequities such as inadequate housing, water, and sanitation. This review aimed to assess housing-related opportunities and challenges during the COVID-19 pandemic among urban poor in low-and middle-income countries. For study identification, a comprehensive search was performed in 11 databases that yielded 22 potential studies. The inadequate housing infrastructure makes the lives of the urban poor more precarious during COVID-19. Typically, the houses lacked lighting, ventilation, and overcrowding. This review reflected that it is crucial to reimagine housing policy for the urban poor with an emphasis on pandemic/epidemic guidelines.

## Introduction

Socioeconomic factors, such as the location of the residence and the housing infrastructure, as well as health-related behaviors, are among the most significant determinants of human health (1, 2). According to the World Health Organization (WHO), improved housing with access to water and sanitation, as well as affordable preventive and curative health care at the doorstep, can empower marginalized groups and improve the entire community's health (1). The term “housing” refers to a safe living space that facilitates daily activities. United Nations-Habitat estimates that ~40 percent of the world's population will require adequate housing by 2030. While everyone is at risk from hazardous housing, those with low incomes and members of vulnerable groups greatly impacted by structural inequities are more likely to reside in inadequate or insecure housing or be denied a home altogether (3). Unmet housing needs have resulted in informal settlers or unplanned settlements like slums, especially among the urban poor (4).

The United Nation-Habitat defined a slum household as a group of individuals living under the same roof in an urban area that often lacks one or more of the following: durable housing, sufficient living space, security of tenure, sanitation, and infrastructure, and access to improved water sources. The slums result from rapid urbanization with a rise in urban population. Hence, local governments confronted with rapid urbanization cannot address the varying requirements for urban infrastructure to address the needs of the urban poor (5).

Low- and middle-income countries (LMICs), as defined by World Bank are those with gross national income (GNI) per capita less than 12,375 USD (6). With economic distress and rapid urbanization, there are multitude of challenges faced by the residents of LMICs. The poorest people, particularly those who live in slums and are homeless, usually have worse health status than their compatriots in rural areas, despite the fact that wealthier urban dwellers can benefit from the “urban advantage” (7).

The influence of COVID-19 on housing, taking into account the complex impacts of physical distancing and isolation, especially among slum inhabitants was challenging. As a result of the droplet and aerosol transmissions, which both can spread COVID-19, overcrowding has been linked to the spread of infections. There was a 50 percent higher risk of COVID-19 incidence (IRR 1.50, 95 percent CI: 1.38–1.62) and a 42 percent higher risk of COVID-19 mortality (MRR 1.42, 95 percent CI: 1.25–1.61) for every 5 percent increase in the percentage of households with suboptimal housing conditions (8).

The deplorable health of the poor or slum dwellers is attributable to inadequate and overcrowded housing conditions triggered by structural inequities. They are also susceptible to several additional housing-related threats, including hazardous electrical connections, toxic building materials, kitchenettes without ventilation, and hazardous infrastructures, such as inadequate sidewalks (3). In addition, the situation worsened during the COVID-19 pandemic. With limited resources, it is difficult for them to implement physical distance and isolation measures. Thus, in purview of the aforementioned mentioned housing related-problems among urban poor, it mandates to conduct research on how the unprecedented events like COVID-19 worsened the situation. Consequently, this review aimed to evaluate and assess housing-related opportunities and challenges during the COVID-19 pandemic among urban poor residing in low- and middle-income countries (LMICs).

## Methods

### Protocol and search strategies

The study protocol of this review is registered in PROSPERO (CRD42022300387). We conducted a comprehensive search to identify studies from the databases–PubMed (MEDLINE), Embase, Web of Science, WHO Global Index Medicus, Epistemonikos, ProQuest, EBSCO, Cochrane, MedRxiv and BioRxiv, 3ie, and Google Scholar for the relevant articles published from November 2019 till August 2021.

### Inclusion exclusion criteria

The studies with slum-dwellers or homeless populations from urban areas of the LMICs as participants were included. Studies with housing-related interventions in COVID-19 the context were included. As we did not look for the effectiveness of the interventions, thus we did not consider any comparator in the included study. The housing-related studies in the context of COVID-19 were included. Any primary research viz. Randomized Controlled Trials and Non-Randomized Studies of Interventions, such as cohort studies, case-control studies, controlled before-and-after studies, and interrupted-time-series studies, were included. The study selection was not restricted to the language of the studies. However, we did not include any secondary data analysis, reviews, commentaries, editorials, and primary studies in the non-COVID-19 context.

### Study selection

Two reviewers screened the studies based on the titles and abstracts. Furthermore, full texts of the potential studies were retrieved and reviewed to check their eligibility for selection. We resolved any disagreements during study selection with discussion and mutual consensus with the other reviewers. The study selection was done independently and in duplicate.

### Quality assessment of the included studies

The quality assessment of the included articles was assessed independently and in duplicate by reviewers based on the criteria mentioned in MMAT (Mixed Methods Appraisal Tool) (9). The disagreements were resolved through discussion and mutual consensus with the other authors. The quality assessment was conducted independently and in duplicate. Methodologically, 9 of 22 studies were in concordance with the MMAT criteria for quality appraisal. The remaining included studies (13 of 22) were of compromised quality, as they deviated and did not fulfill the required criteria of the MMAT tool.

### Data extraction and synthesis

Two reviewers extracted data in a Microsoft Excel sheet template comprising study characteristics (author, year, country, study design, sample size, study setting, data collection methods), and results (outcome measures, conclusion, recommendations. We resolved the disagreements at any stage *via* discussions with the authors. Furthermore, the author (KCS) reviewed all the studies, open-coded the information, and prepared a codebook—a thematic framework that emerged from the data for selective coding in MAXQDA Analytics Pro 2020 (VERBI GmbH, Berlin). The author SD extracted all the information in MAXQDA using the selective coding approach. Finally, the authors (SD, KCS) synthesized and prepared the results using thematic framework analysis.

## Results

**Figure 1:**
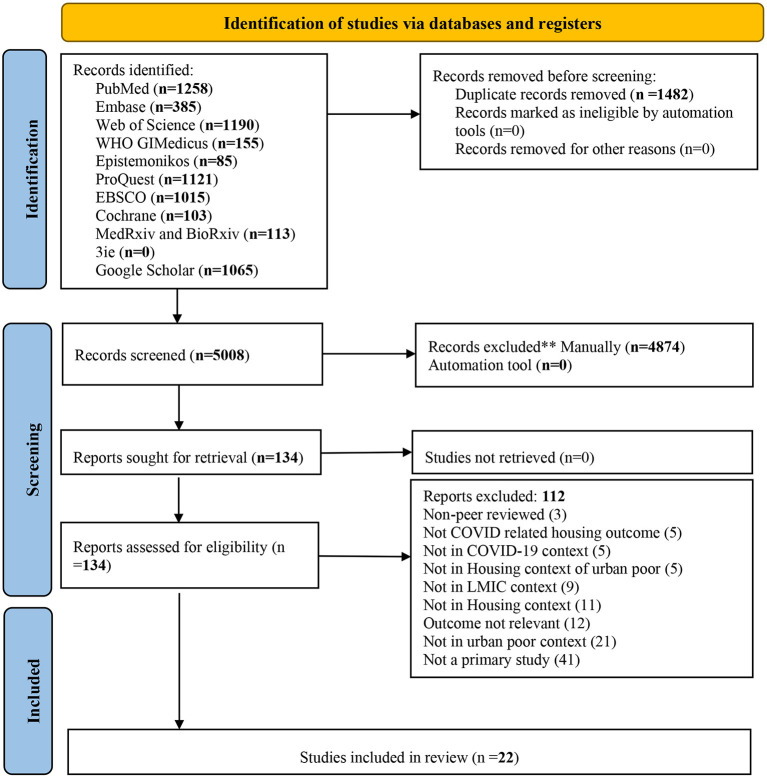
PRISMA flow diagram (10).

We identified 6,490 studies, including 1,482 duplicates. Based on the title and abstract, 5,008 studies were screened, resulting in 134 potential studies for full-text review. Out of 134, 22 studies met inclusion criteria and were finally included in the review. The PRISMA flow diagram is provided to illustrate the entire study selection process in Figure 1.

### Characteristics of included studies

Of the 22 included studies, nine were qualitative, eight were quantitative, and five were mixed-method studies. Most of the included studies were from India (*n* = 8) followed by Bangladesh (*n* = 6), Nigeria (*n* = 3), South Africa (*n* = 2), Vietnam (*n* = 1), Turkey (*n* = 1), and Mixed (*N* = 1). The urban poor in the included studies comprised refugees, slum dwellers, migrant workers, and the urban homeless (Table 1).

**Table 1:** Characteristics of included studies.

**References**	**Country**	**Urban Poor**	**Population (*N*)**	**Study type**	**Data collection method**	**Data Analysis**	**Result**
Akter et al. (11)	Bangladesh	Refugee	66	Qualitative	In-depth interviews	Thematic analysis	Insufficient built infrastructure and unhygienic living conditions, including improper WASH management, increase their risk of COVID-19.
Akter et al. (12)	Bangladesh	Slum dwellers	42	Qualitative	In-depth interviews	Thematic analysis	Infrastructural, and health-related issues had affected slum dwellers' COVID-time vulnerabilities.
Anand et al. (13)	Vietnam	Migrant workers	31.4 million	Quantitative	Secondary data (PLFS, NSSO)	Not reported	The majority (51%) of the slum population reside in a single room while 45% of single rooms were shared by 3–4 members. Thus, the slum population was densely packed making it difficult to comply with the social distancing norms.
Auerbach et al. (14)	India	Slum	321	Quantitative	Telephonic survey	Descriptive statistics	The housing in slums has dense living conditions making social distancing impractical to some extent. They lacked taps in their homes and rely on communal taps or water tankers.
Bercegol et al. (15)	India	Slum	Not reported	Qualitative	Telephonic In-depth interviews	Not reported	The housing in the refugee camps is orthonormal planned with narrow alleys and without pavement.
Budak et al. (16)	Turkey	Refugees	414	Quantitative	Questionnaire	Descriptive statistics	Tents are more sensitive against pandemics while the ones who stay in pre-fabricated houses, tents, and detached houses have lower levels of combating pandemics.
Bueno et al. (17)	Nigeria	Informal settlement	510	Quantitative	Interviews	Inverse probability reweighing	Basic sanitary infrastructure is lacking
Bui et al. (18)	Nigeria	Urban slum	445	Quantitative	Self-administered questionnaire	Cumulative risk assessment	About two third of the migrant workers lived in a small houses (< 36 m^2^) with their families.
Cloete et al. (19)	South Africa	Sex workers and homeless	60	Qualitative	Informant interview, Focus Group Discussion	Thematic analysis	Closely-packed houses and shacks in informal settlements make physical distancing impractical. The spatial organization of the slum settlements makes it unconducive to maintain social distancing.
Ebekozien et al. (20)	Nigeria	Informal settlement	40	Qualitative	In-depth interviews	Thematic analysis	The majority of informal settlements spread COVID-19 as the dwellings were densely packed with a dimension lesser than 4 feet against the minimum standard of 6 feet with inadequate basic amenities, making individual or group quarantine difficult.
Enwerekowe et al. (21)	Bangladesh	Urban slum	Not reported	Mixed-method	Structured and non-structured interview	Photograph and narratives	Overcrowding, multi-generational homestead composition, extreme poverty, and unchecked mixed-usage of residential spaces as major challenges to effective social distancing (and self-isolation).
Gibson et al. (22)	Bangladesh	Informal settlement	Not reported	Quantitative	GIS Data analysis	Descriptive statistics	The dwellings are denser in fashion making it impractical to practice social and physical distancing while staying at home or outside in the selected settlements.
Hasan et al. (23)	Bangladesh	Urban slum	588	Quantitative	Survey	Exploratory and secondary analysis of World Bank data	Most households were single-room dwellings (80.4%). Median crowding ranged from 0.55 m^2^ per person up to 67.7 m^2^ per person. A significant positive relationship between crowding and the use of shared facilities.
Mohan et al. (24)	India	Slum	113	Mixed-method	Interviews	Descriptive statistical analysis	Pre-existing vulnerabilities (lack of safe drinking water, decent housing, and sanitation) of the workers have amplified and become more visible during the recent COVID-19 pandemic.
Napier-Raman et al. (25)	India	Slum	122	Mixed-method	The rapid survey, in-depth semi-structured interview.	Descriptive and Thematic analysis.	Housing insecurity was a crucial effect of the lockdown, with no clear government policy on rent waiving affecting many families.
Nyashanu et al. (26)	South Africa	Informal settlement	30	Qualitative	In-depth interviews	Interpretive phenomenological analysis	Overburdened infrastructure in the informal settlement, lack of savings- loss of income and shortage of food, anxiety depression made it difficult to practice social distancing.
Patel (27)	India	Slums	Not applicable	Mixed-method	Media reports analysis	Quantitative analyses	When rooms are shared by multiple family members (overcrowding) and used for multiple purposes, extremely challenging to follow home quarantine.
Sahu and Dobe (28)	India	Slum	Not applicable	Qualitative	Document analysis	Document analysis	Overcrowding without provision for safe drinking water, sanitation, or other basic services, make the life of the urban poor challenging.
Saldanha (29)	India	Slum	1	Qualitative	Narrative	Self-reported	Crowding and lack of running water made it difficult to practice social distancing and hygiene among residents.
Spiritus-Beerden et al. (30)	Global	Refugees and migrants	20,742	Quantitative	online survey	Descriptive and exploratory factor analysis	The mental health of refugees and migrants during the COVID-19 pandemic was significantly impacted particularly by insecure housing situations and residence status
Wasdani et al. (31)	India	Slum	6	Qualitative	Case study	Self-reported	The slum's close quarters and communal areas served as ideal breeding grounds for a virus that spread through close physical contact.
Williams et al. (32)	Bangladesh	Urban poor	525	Mixed-method	Telephonic interview	Descriptive statistics	Overcrowding made it difficult to practice preventive measures.

Three main themes emerged: (1) Housing infrastructure and existing facilities, (2) Challenges related to housing conditions during COVID-19 pandemics, and (3) Coping mechanisms, social support, and expectations.

### Theme 1: Housing infrastructure and existing facilities

#### Usual population density of the households and community

The poor urban environment's unplanned housing and spatial organization make it unconducive to a healthy lifestyle. In general, it was reported that the dark and unventilated houses with leaky roofs and damped walls increased the vulnerability of the residents. The non-paved narrow lanes measuring about 1 to 1.25 meters with large gatherings contribute to life-threatening congestion while exposing residents to airborne virus transmission. These congested lanes contribute to insufficient indoor lighting and ventilation. As far as crowding is concerned, 4–5 individuals dwell in houses with dimensions ranging from 9.29 to 13.243 meters square. They are bound to improvise a wall in their small rooms to create a living and bedroom. It is challenging for the slum residents to maintain their temporary dwellings as they face adversity due to heavy rain and flooding. The housing units lacked connectivity to basic sanitation infrastructure (11–14, 17, 28). In refugee camps, houses were orthogonal and normal in 10 square feet. These houses were made of concrete and tin with slender and unpaved pathways provided with free electricity and water (15). Most housing units failed to meet the minimum housing lobby standard of 6 feet, as they were even smaller than 4 feet (20).

The study revealed that the houses were constructed in rows with rooms ranging from 80 to 100 square feet. These tiny houses were permanent, with shared bathrooms and metered water taps set at a distance. Twenty families were forced to share a single bathroom and three toilets, which was a clear indication of the facility's high loads. The housing alleys were crowded with diverse populations (24). In slums, an average of 3.2 people resided in a single room, but in non-slums, 2.9 people resided in a single room. In slums, 1.7 rooms were shared by 4.7 people. The majority of urban slum dwellings lacked separate kitchens. Cooking and sleeping in the same room made maintaining physical distance difficult (27). Most slum residents who lack housing amenities reside on sidewalks, putting their health and way of life at risk (30, 32). According to the data, a little shelter was filled by 10 to 11 people, which equates to 1.6 to 2.9 square meters per person, which is below the Humanitarian Charter's suggested standard of 4.5 square meters per person (11–13, 23).

#### Lack of living space and internal WASH facilities in household

Along with the lack of living spaces, the urban slum residents encountered other problems: they didn't have access to safe and sufficient water facilities for hygiene practices in their households. Most of these cohorts relied on ordinary communal tap water, borewells, or water tankers for their water needs (14). The slum residents had to share toilets and water sources to carry out their daily chores. It is evident from the findings that crowding is significantly high in households sharing both bathing and toilet facilities (median=2.9 m^2^/person, 95% CI, 2.63–2.93) (23). Typically, eight to 10 individuals share a tiny 150 sq. ft. shack without natural light or ventilation and no access to safe drinking water, sanitation, or other basic amenities. They did not have separate toilet facilities in their homes and had to rely on dirty, unhealthy, and risky communal lavatories. Moreover, it was more challenging for maintaining hygiene among women and girls. They were even bound to urinate and defecate in the open. They lacked a kitchen in their households. They even had to leave their homes to fetch water from communal taps or tube wells, thus risking infection. Restricted and timed water supplies make it impractical to practice hand hygiene (28).

### Theme 2: Challenges related to housing conditions during COVID-19 pandemics

#### Major challenges during COVID-19

There were many challenges faced by the urban poor due to poor housing conditions. Temporary refugee camps in the lowlands make them more vulnerable to seasonal flooding and calamities like COVID-19. Poor infrastructure and crowding make it challenging to practice physical distancing or follow quarantine measures (11–13, 22). As a respondent stated:

“*We cannot maintain physical distance within and outside our shelter. Due to the roofing materials, tarp, insufficient sun fans, and natural ventilation, our room becomes extremely warm during this scorching summer. Due to the overcrowding and the risk of contracting Coronavirus, we were unable to remain in a room for an extended period.”* *(**11**)*

Migrant communities reported struggling to make their living and pay housing rents, which were further aggravated, by food insecurity and stringent lockdown during the COVID-19 infection (19). Lack of apparent government policy concerning rent waiver, many families faced housing insecurity due to the lockdown (25). The anger and outrage among the informal settlements were marked by the Government's impractical policy toward COVID-19 containment (20). As quoted by the respondents

“*They [Government] are talking about social distancing and regular handwashing, please ask them if there is a water supply for regular handwashing. Many of the houses in this neighborhood have challenges with toilets, water supply, and adequate housing to the best of my knowledge. So, how can I comply with the physical distancing of about six feet apart when my next neighbor is directly opposite my room with a lobby of about three to four feet wide and shared facilities such as toilet, kitchen, bathroom, etc.?”* *(**20**)*

Abiding by the physical distancing norms was a major challenge among the poor urban cohort as they had to deal with overcrowding and homestay orders (20, 21). Due to overcrowding, many inhabitants were compelled to seek refuge under trees and in makeshift outdoor seating places (21). The housing and basic amenities were not optimal, and the rent-seeking mechanism was unjustified. Women had no safe location to meet and socialize, and children had no space to play due to crowded areas, thus deteriorating their health and living with time (24). COVID-19 left many homeless slum dwellers to move into makeshift shelters as the administration forcibly moved them from their usual sleeping spaces (32).

#### Increase in usual population density, challenges in physical distancing, and overcrowding associated with infection

The overcrowding worsened during COVID-19 as the slum infrastructures were not conducive to maintaining physical distancing and abiding by the quarantine norms due to lockdown enforcement (11, 12). The houses were not optimally apart in slums; they depended on a common water source. Overcrowding was not just confined to homes; it was even seen in minibuses or taxis, making physical distancing among informal settlers impossible (19). The rented house was also overcrowded during COVID-19 due to lockdown and movement restrictions imposition, making it challenging to practice physical distancing, resulting in fear and anxiety of catching an infection (21, 22, 26). Furthermore, women in slums fared worse than their male and female non-slum counterparts in terms of access to secure housing for isolation and physical distance. Most slum women work, but it is usually low-paying, transitory, and exploitative, making them more likely to perform poorly. Many domestic workers could not provide services remotely, resulting in a loss of income that allowed them to feed their families. The concern over the impracticality of taking containment measures among the slum residents was quite evident from the respondents' statements.

“*I cannot imagine how residents would practice social distancing and hygiene, given crowding and the lack of running water.”* *(**29**)*

“*Sadly, the tight quarters and the communal spaces of the slums are natural conduits for a virus that relies on physical closeness to spread.”* *(**31**)*

#### Challenges concerning isolation–social conflict and stigma

Any crisis comes with loads of challenges. The cramped housing spaces (both indoor and outdoor) make it difficult for refugees to adhere to COVID-19 restrictions as prescribed. The same was evident from the stated statement by a female respondent.

“*We cannot maintain social distancing in our shelter and outside as well. Our room becomes very hot during this hot summer due to the roofing materials, tarpaulin, insufficient solar fans, and natural ventilation. Therefore, we cannot stay in a room for long, and can't stay outside also because of overcrowded people and the risk of Coronavirus.”* *(**11**)*

The slum's spatial layout and infrastructural instability made it difficult to maintain social distance. Around 16–30 households share one toilet/bath, while 25–30 families share a tube well. Similarly, about 20 people who had access to water with compromised water quality shared a tap. The lanes in the urban poor residential area were so compact that they found it very difficult to move. The dense living conditions are the biggest challenge in maintaining physical distancing norms (11, 12, 14, 19). A resident stated

“*The lanes are so narrow that we can barely cross each other; we rub shoulders...We need to travel outside to use common toilets…It is hard to maintain quarantine as we don't have sufficient space and lack (attached) toilets …”* *(**12**)*

The poor urban residents felt stigmatized for contracting the infection due to their overcrowded living conditions. Apart from making it impractical to practice physical distancing, they were under threat of getting a disease as a shack measuring 6 to 15 meters square was shared by around 10 individuals (26). The poor and compacted housing infrastructure significantly affected the anxiety level and exacerbated levels of gender-based violence (16).

### Theme 3: Coping mechanism, social support, and expectations

#### Mal-adaptive practices

Due to the enforcement of stringent lockdowns, physical distancing was hindered as the residents with staggering work in the poor urban regions were compelled to stay at home; consequently, overcrowding worsened more. Insufficient spacing and overcrowding lead residents to take refuge under trees or opt for makeshift seating spaces outdoors (21). Deterioration in health conditions was reported among the residents with the insecure residence. Furthermore, people residing in perilous environments like asylum, streets, or insecure areas were experiencing daily stressors, discrimination along with degrading in their mental health (30).

#### Social support and community members' expectations

The crises dealt with community engagement and involvement. The rural childcare center, educational institution, community center, and train coaches were transformed into isolation centers to ensure physical distancing (15). Migrant workers were supported with personal protective equipment, food, and housing by the company (18). Investing in decent, affordable, and resilient housing needs was to be prioritized as it could deliver healthcare and prosperity to the individuals.

## Discussion

The findings demonstrated that the inadequate housing infrastructure makes the lives of the urban poor more precarious during unprecedented events such as COVID-19. Typically, the houses lacked enough lighting and ventilation. The overcrowding was visible, as the houses and camps in urban poor areas had disproportionate dimensions and clogged streets. The quality of the homes was so low that they could barely withstand harsh conditions such as rain and flooding.

Throughout COVID-19, housing with inadequate WASH infrastructure in urban poor settlements posed a difficulty. Typically, this population lacked personal bathrooms and water taps, requiring them to rely on unreliable communal or shared water supplies and community toilets. During pandemics, this deficiency makes it more challenging to adhere to infection control standards such as physical separation, hand hygiene, and isolation. In a few instances, planned housing with amenities for the urban poor was reported; however, the facilities were sadly shared. According to the results, a handful of the urban poor did not have the luxury of four walls and a roof and was forced to sleep on the streets. The results also indicate that overcrowding worsened due to the lockdown, as the restricted movement made it difficult to adhere to physical distance restrictions. With the increase in population, the stigma of catching the infection increased inside and beyond the home. This increased domestic violence, a problem regardless of gender, as working family heads were forced to remain at home (33–35). Frequently, women in urban slums experience violence (36). Their lives are endangered by maladaptive behaviors like sheltering on or beneath trees.

According to the facts, there was very little social or communal support. However, a temporary effort was made by converting train coaches or institutions into isolation centers. The community's expectations for basic facilities from the government were not realized. Housing insecurity is widespread in LMICs, particularly in urban slums where many urban residents live. According to the data, housing-related problems increased among urban poor inhabitants during unanticipated pandemics. The inclusion of just studies from LMICs limits the generalizability of the findings. As the inequalities and discrepancies among the urban poor regarding access to housing and essential utilities become apparent amid this horrific pandemic, it serves as a wake-up call for concerned authorities worldwide.

There is an urgent need to scale up the implementation of initiatives such as Prime Minister Housing Scheme and JAGA mission of India, Housing microfinance and Participatory Slum Upgrading Program (PSUP) of African countries, Argentina's Slum Housing Upgrade Program across all LMICs. These housing schemes provided accommodation for the urban poor or slum dwellers living in cramped conditions with inadequate infrastructure, hygiene, and drinkable water, thereby improving their quality of life and health (37–42). These are based on *in-situ* slum redevelopment concept that uses slum-occupied land to incentivize the creation of formal settlements for slum residents by private actors. Researchers and policymakers must comprehend and address the housing needs of this vulnerable population. There is little evidence of the impact of housing infrastructure on the overall health of the urban poor. In the context of LMICs, understanding the problem of slum growth and urban housing shortage is crucial. As slums grow as a result of systemic failure, their transformation necessitates a combination of political will, committed leadership, and empowered communities to enable urban changes that are consistent and inclusive.

The incidence and effects of COVID-19 can vary substantially across space and time, with urban populations initially being severely affected. Effective support for poor and vulnerable households will require substantial additional financial resources. In addition, the older adults, children, disabled, and women are prone to have the worst effects. The COVID-19 pandemic is wreaking havoc on women's health and social and economic well-being worldwide. As the pandemic expands to LMICs, the response must address the underlying injustices that put women and girls at greater risk in slums. Women also bear a disproportionate share of the load at home due to school and childcare facility cutbacks, as well as long-standing gender disparities in unpaid labor. During times of crisis and quarantine, women suffer heightened risks of employment and income loss and increased dangers of violence, exploitation, abuse, or harassment. Policy solutions must be quick and take into account the concerns of women (43, 44). Fundamentally, all policy responses to the crisis must incorporate a gender lens and consider women's distinct demands, responsibilities, and viewpoints.

It is crucial to reimagine housing policies for the urban poor, particularly in LMICs, emphasizing pandemic and epidemic management protocol. The COVID-19 Pandemic highlights and exacerbates existing structural inequities and endangers the lives of the urban poorest segments of the population. The pandemic criteria did not align with existing housing, water, and sanitation facilities. The urban slum encountered many obstacles, but in light of the gravity of the situation, they overcame them with patience and diligence. Urban planning and policies should also ensure that the urban poor's housing infrastructure complies with basic housing and health criteria. Urban areas demand a healthy and sustainable housing strategy for the urban poor. Along with other essential utilities, the urban poor should be granted housing rights on humanitarian grounds. A time-bound plan is necessary to support the envisioned urbanization program.

## Author contributions

KS, SD, and GD developed the protocol. SD and MS completed the search, screened the articles for inclusion, extracted the data, and completed the risk of bias assessments. KS, PM, SD, and GD extracted the data and synthesized the findings, interpreted the results, and drafted the manuscript. SP and MB interpreted the results. All authors critically revised the manuscript. All authors approved the final version.

## Funding

This systematic review was funded by the UNICEF/UNDP/World Bank/WHO Special Programme for Research and Training in Tropical Diseases (TDR), World Health Organization, Geneva, Switzerland (Grant No: 2021/1086892-1/P20-00116).

## Conflict of interest

The authors declare that the research was conducted in the absence of any commercial or financial relationships that could be construed as a potential conflict of interest.

## Publisher's note

All claims expressed in this article are solely those of the authors and do not necessarily represent those of their affiliated organizations, or those of the publisher, the editors and the reviewers. Any product that may be evaluated in this article, or claim that may be made by its manufacturer, is not guaranteed or endorsed by the publisher.

## Author disclaimer

The authors alone are responsible for the views expressed in this article and they do not necessarily represent the views, decisions, or policies of the institutions with which they are affiliated.
